# Supplementary material for: Flow Simulation and Gradient Printing of Fluorapatite- and Cell-Loaded Recombinant Spider Silk Hydrogels
Source: Biomolecules. 2022 Oct 3;12(10):1413. doi: 10.3390/biom12101413 (PMC9599405; doi:10.3390/biom12101413)
Supplement: Supplementary file 1 [file biomolecules-12-01413-s001.zip › biomolecules-1907633-supplementary.pdf]

biomolecules

MDPI

Supplementary Information

Flow Simulation and Gradient Printing of Fluorapatite and Cell loaded Recombinant Spider Silk Hydrogels

Vanessa J. Neubauer[1], Florian Hüter[2], Johannes Wittmann[2], Vanessa T. Trossmann[1], Claudia Kleinschrodt[2], Bettina Alber-Laukant[2], Frank Rieg[2,3,4] and Thomas Scheibel*[1,5,6,7,8]

[1] Lehrstuhl Biomaterialien, Fakultät für Ingenieurwissenschaften, Universität Bayreuth, Prof.-Rüdiger-Bormann-Straße 1, 95447 Bayreuth, Germany
[2] Lehrstuhl Konstruktionslehre und CAD, Fakultät für Ingenieurwissenschaften, Universität Bayreuth, Universitätsstraße 30, 95440 Bayreuth, Germany
[3] Bayreuth Engine Research Center (BERC), Universität Bayreuth, Universitätsstraße 30, 95440 Bayreuth, Germany
[4] Zentrum für Energietechnik (ZET), Universität Bayreuth, Universitätsstraße 30, 95440 Bayreuth, Germany.
[5] Bayreuther Zentrum für Kolloide und Grenzflächen (BZKG), Universität Bayreuth, Universitätsstraße 30, 95440 Bayreuth, Germany
[6] Bayerisches Polymerinstitut (BPI), Universitätsstraße 30, 95440 Bayreuth, Germany
[7] Bayreuther Zentrum für Molekulare Biowissenschaften (BZMB), Universität Bayreuth, Universitätsstraße 30, 95440 Bayreuth, Germany
[8] Bayreuther Materialzentrum (BayMAT), Universität Bayreuth, Universitätsstraße 30, 95440 Bayreuth, Germany
* Correspondence: TS thomas.scheibel@bm.uni-bayreuth.de

Citation: Neubauer, V.J.; Hüter, F.; Wittmann, J.; Trossmann, V.T.; Kleinschrodt, C.; Alber-Laukant, B.; Rieg, F.; Scheibel, T. Flow Simulation and Gradient Printing of Fluorapatite and Cell loaded Recombinant Spider Silk Hydrogels. *Biomolecules* **2022**, *12*, 1413. https://doi.org/10.3390/biom12101413

Academic Editors: Jayakumar Rajadas and Mustafeez Mujtaba Babar

Received: 25 August 2022
Accepted: 27 September 2022
Published: 3 October 2022

Publisher's Note: MDPI stays neutral with regard to jurisdictional claims in published maps and institutional affiliations.

Copyright: © 2022 by the authors. Licensee MDPI, Basel, Switzerland. This article is an open access article distributed under the terms and conditions of the Creative Commons Attribution (CC BY) license (https://creativecommons.org/licenses/by/4.0/).

Content on pages S2-S4

Figure S1. Simulation model.
Figure S2. Gradient printing pre-studies.
Figure S3. Rheology.
Figure S4. Fluorapatite characterization.
Figure S5. DIN EN ISO 10993-5 results for particle species.
Additional Information: **Video S1**. Flow Simulation.

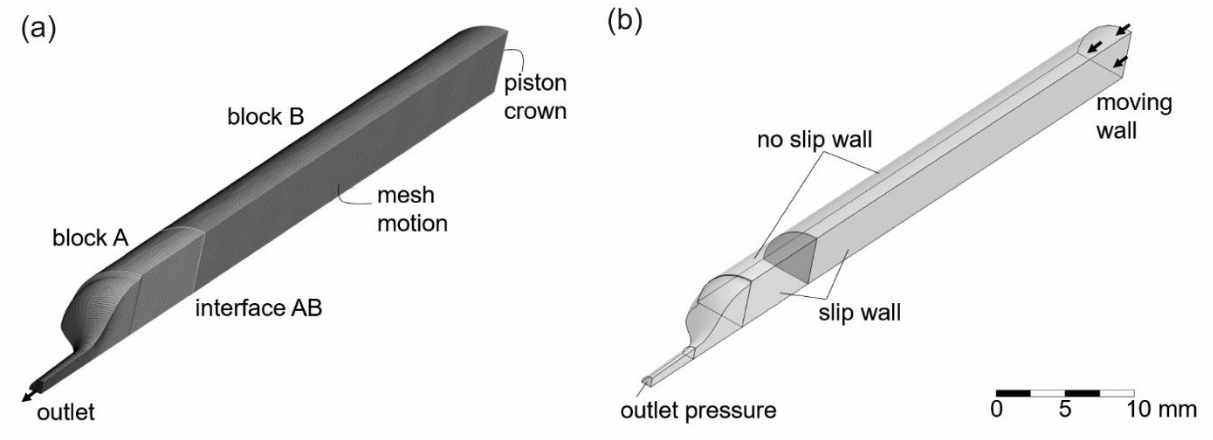

Figure S1. Cartridge model for the simulation of the a) AB block-system and mesh as slip wall and b) AB block boundary conditions.

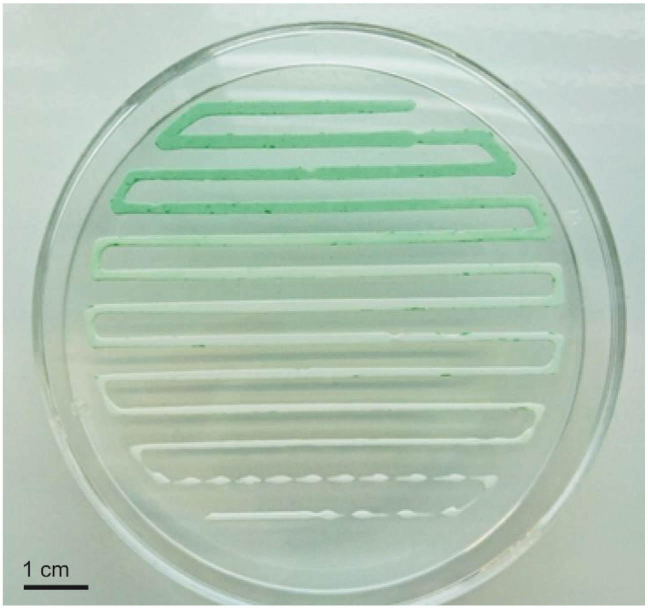

Figure S2. Photograph of 3D gradient printing results with coloured water-in-oil emulsion as an exemplary AB block system.

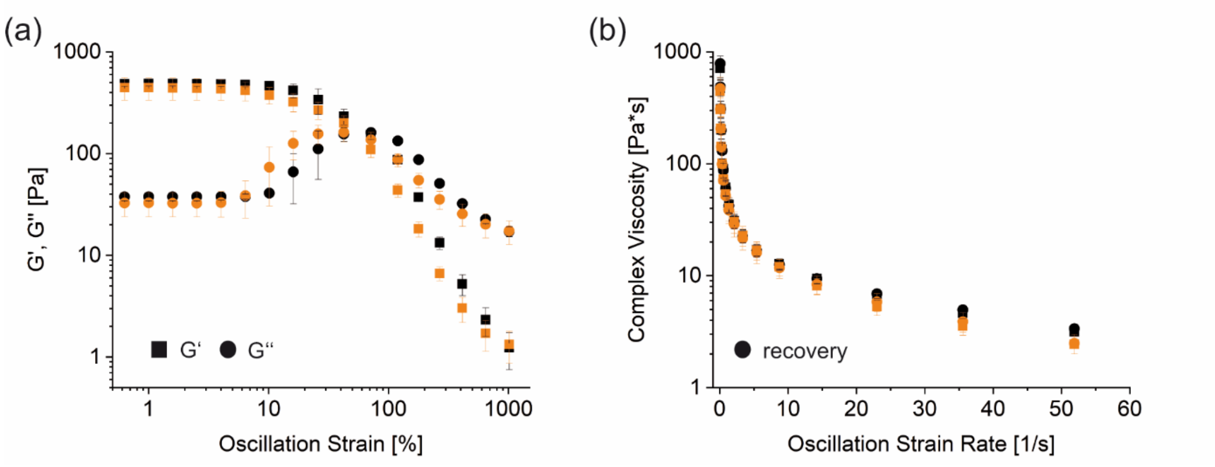

Figure S3. Rheological characterization of 3 % w/v eADF4(C16) (black) and eADF4(C16)/FITC-eADF4(C16) (orange) hydrogels: a) Mean amplitude sweep measurements with yield points at the G′ and G′′ cross-over. b) Mean frequency sweep measurements showing shear-thinning behaviour and recovery.

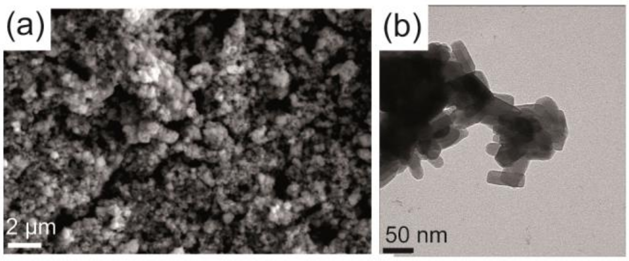

(c)

Atomic ratio [%]	Synthesized FAp	Stoichiometry FAp
Ca:F	5.117	5
Ca:P	1.633	1.667
Ca:O	0.503	0.417

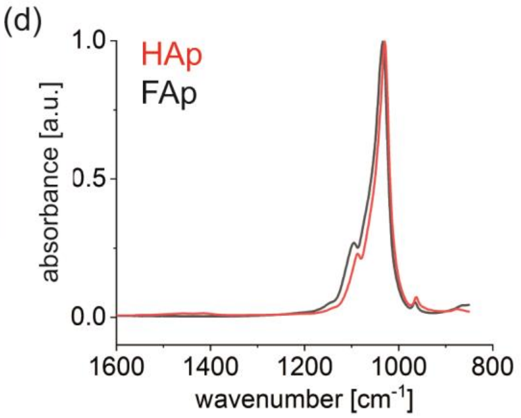

Figure S4. Characterization of fluorapatite (FAp) particles: a) SEM image of dry FAp particles. b) TEM images of FAp particles. c) SEM-EDX analysis derived atomic ratio of typical elements in FAp compared to its stoichiometry in %. d) Mean ATR-FTIR spectra overlay of synthesized FAp particles (black) with that of commercially available hydroxyapatite particles (red) as reference.

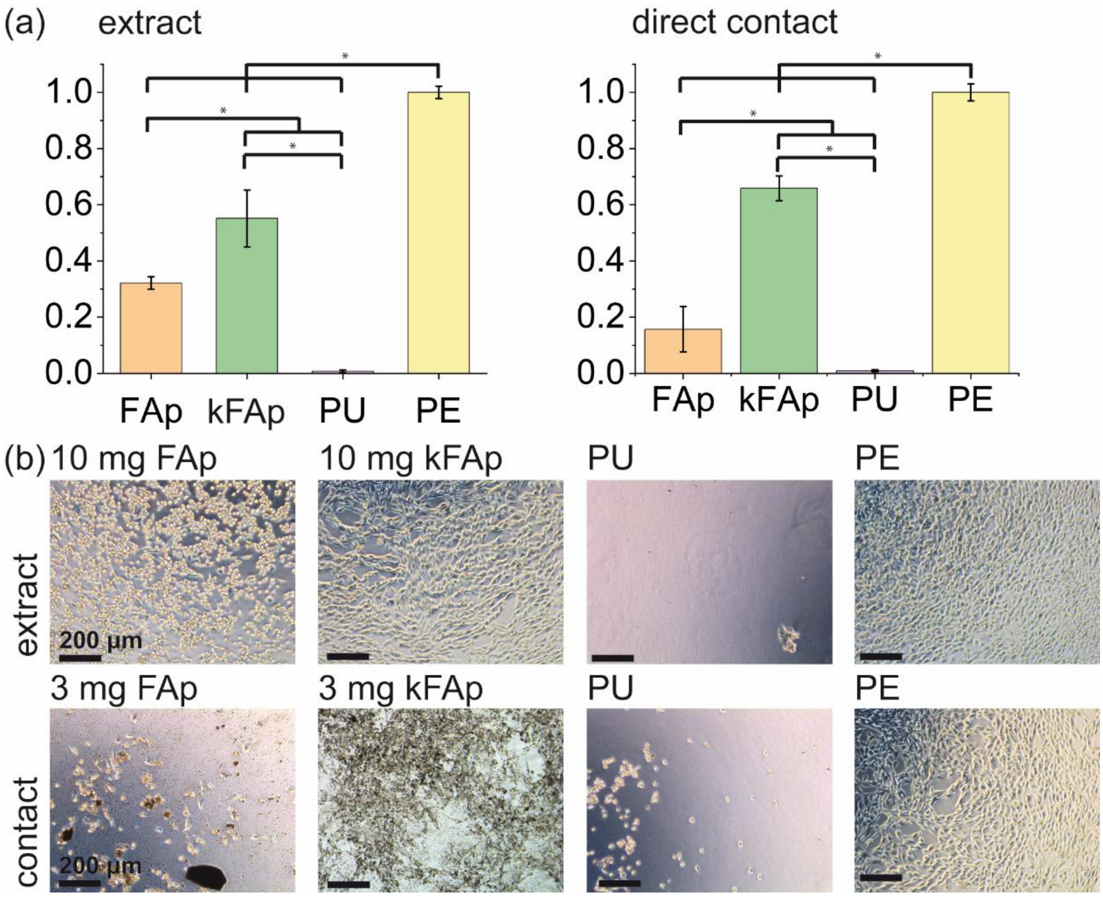

Figure S5. Cytotoxicity evaluation of uncoated (FAp) and eADF4(κ16)-coated (κFAp) fluorapatite particles according to DIN EN ISO 10993-5. a) Quantified viability using Cell Titer Blue assay and b) cell morphology of BALB/3T3 fibroblasts upon direct contact or upon contact with an extract of particles. High density polyethylene served as positive and organotin-stabilized polyurethane as negative control for extract and direct contact test (n=3 for all conditions). Extract and direct contact tests were analysed using light microscopy, scale bar 200 µm. κFAp refers to FAp particles coated with eADF4(κ16).
